# Effect of Mutant and Engineered High-Acetate-Producing *Saccharomyces cerevisiae* var. *boulardii* Strains in Dextran Sodium Sulphate-Induced Colitis

**DOI:** 10.3390/nu16162668

**Published:** 2024-08-13

**Authors:** Sara Deleu, Inge Jacobs, Jorge F. Vazquez Castellanos, Sare Verstockt, Bruna Trindade de Carvalho, Ana Subotić, Bram Verstockt, Kaline Arnauts, Lowie Deprez, Eva Vissers, Matthias Lenfant, Greet Vandermeulen, Gert De Hertogh, Kristin Verbeke, Gianluca Matteoli, Geert R. B. Huys, Johan M. Thevelein, Jeroen Raes, Séverine Vermeire

**Affiliations:** 1 TARGID, Department of Chronic Diseases and Metabolism (CHROMETA), KU Leuven, 3000 Leuven, Belgium; sara.deleu@kuleuven.be (S.D.); eva.vissers@kuleuven.be (E.V.);; 2 VIB-KU Leuven Center for Microbiology, 3001 Leuven, Belgiumgeert.huys@kuleuven.be (G.R.B.H.);; 3 Department of Microbiology, Immunology and Transplantation, Rega Institute, KU Leuven, 3000 Leuven, Belgium; 4 NovelYeast bv, Bio-Incubator BIO4, Gaston Geenslaan 3, Leuven-Heverlee, 3001 Leuven, Belgium; 5 Department of Gastroenterology and Hepatology, UZ Leuven, KU Leuven, 3000 Leuven, Belgium; 6 Laboratory of Morphology and Molecular Pathology, UZ Leuven, 3000 Leuven, Belgium

**Keywords:** ulcerative colitis, microbiota modulation, probiotics, *Saccharomyces*, acetate, SCFA

## Abstract

Acetate-producing *Saccharomyces cerevisiae* var. *boulardii* strains could exert improved effects on ulcerative colitis, which here, was preclinically evaluated in an acute dextran sodium sulphate induced model of colitis. Nine-week-old female mice were divided into 12 groups, receiving either drinking water or 2.75% dextran sodium sulphate for 7 days, combined with a daily gavage of various treatments with different levels of acetate accumulation: sham control (phosphate buffered saline, no acetate), non-probiotic control (Baker’s yeast, no acetate), probiotic control (Enterol^®^, transient acetate), and additionally several *Saccharomyces cerevisiae* var. *boulardii* strains with respectively no, high, and extra-high acetate accumulation. Disease activity was monitored daily, and feces samples were collected at different timepoints. On day 14, the mice were sacrificed, upon which blood and colonic tissue were collected for analysis. Disease activity in inflamed mice was lower when treated with the high-acetate-producing strain compared to sham and non-probiotic controls. The non-acetate-producing strain showed higher disease activity compared to the acetate-producing strains. Accordingly, higher histologic inflammation was observed in non- or transient-acetate-producing strains compared to the sham control, whereas this increase was not observed for high- and extra-high-acetate-producing strains upon induction of inflammation. These anti-inflammatory findings were confirmed by transcriptomic analysis of differentially expressed genes. Moreover, only the strain with the highest acetate production was superior in maintaining a stable gut microbial alpha-diversity upon inflammation. These findings support new possibilities for acetate-mediated management of inflammation in inflammatory bowel disease by administrating high-acetate-producing *Saccharomyces cerevisae* var. *boulardii* strains.

## 1. Introduction

The human gut microbiome plays an important role in health and disease [1]. Several disorders, including inflammatory bowel disease (IBD), have been linked to changes in the gut microbiota composition [2]. For both Crohn’s disease (CD) and ulcerative colitis (UC), the two main clinical phenotypes within IBD, a decrease in α-diversity and species richness, as well as an increase in the prevalence of the inflammation-associated *Bacteroides2* enterotype, compared to controls has been observed [3,4]. In terms of bacterial abundances, a reduction of specific taxa with key metabolic properties such as *Bifidobacterium* spp. and *Clostridium* Groups IV and XIVa, including *Faecalibacterium prausnitzii* and *Roseburia* spp., together with a relative increase in bacteria possessing pro-inflammatory properties like *Pasteurellaceae*, *Escherichia coli*, and *Fusobacteriaceae* have been demonstrated in IBD patients [5,6,7,8].

Modulation of the gut microbiome composition to treat or improve quality of life of IBD patients is being studied intensively [9]. While fecal microbial transplantation studies have shown promising results in open label and placebo-controlled trials [10,11,12,13], success rates seem donor- and recipient-dependent [14]. The use of well-defined probiotics is an alternative option that does not rely on donor availability and poses less concerns regarding unwanted side effects. For instance, the yeast *Saccharomyces cerevisiae* var. *boulardii* [e.g., Enterol^®^, Biocodex, Gentilly, France] might be used as a supportive treatment to reduce relapses in IBD patients [15]. A recent randomized, double-blind, placebo-controlled trial observed decreased intestinal inflammation in patients with asymptomatic UC and CD upon multi-strain bacterial probiotic treatment [16].

Another potential therapeutic strategy is based on the observed alterations in fecal microbial metabolite concentrations [17], including a reduction in short-chain fatty acids (SCFAs) and dysregulation of bile acid derivatives and tryptophan metabolites in IBD patients [3,17]. SCFAs such as acetate and butyrate have therefore gained interest for their potential beneficial effects in IBD [18]. Most SCFAs studies have focused on butyrate in which beneficial effects on gut microbiome composition, intestinal barrier function, and inflammation were reported [19]. However, in the tested concentration range of 3–8 mM, butyrate has also been shown to have a toxic effect on epithelial colon cells in vitro, especially in the presence of an altered mucus layer which is often the case in UC [20]. Although the mode-of-action is less understood, acetate is interesting for modulation purposes due its lower toxicity to epithelial cells and its potential to support the growth of butyrate-producing bacteria by metabolic cross-feeding [21]. Moreover, a recent report suggested that the probiotic potential of *Saccharomyces cerevisiae* var. *boulardii* (Sb) might be related to its unusually high acetic acid production of up to 100 mM in mutant strains [22].

In our previous work, beneficial effects of high acetate concentration (100 mM) were observed in vitro on organoid-derived epithelial monolayer cultures from UC patients. Therefore, the aim of this preclinical study was to evaluate genetically modified Sb strains with different levels of acetate production in a dextran sodium sulphate (DSS)-induced mice model of colitis. The objectives of the study were to evaluate the effects of genetically modified Sb strains on intestinal inflammation, secretion of serum cytokines, and fecal short chain fatty acid concentrations. We also studied if different levels of acetate production by these genetically modified mice were associated with changes in gene expression and microbiome composition.

## 2. Materials and Methods

Animals, ethical approval, sample size, and randomization: Ethical approval was obtained by the KU Leuven ethical committee for animal experimentation with P090/2020. The mice [C57/Bl6 JAX] were ordered from Charles River. Sample size was calculated based on an effect size of 0.5, alpha of 0.05, and power of 80%, and after adjusting accordingly to buffer for potential mortality, a group size of 10 was implemented. Before the experiments were initiated, the mice were acclimatized for at least two weeks in which they were handled to reduce stress-related bias, and the mice were randomized over the treatment groups based on their weight using RandoMice. 

*Saccharomyces cerevisiae* var. *boulardii* strains: The *Saccharomyces* strains (Table 1) were in-house produced by the Thevelein lab (Novelyeast, Leuven-Heverlee, Belgium) as previously described [23]. After production the strains were prepared for gavage by dissolving them in 2 mL PBS (2 × 10^9^ CFU/mL) containing 30% glycerol.

**Figure 1:**
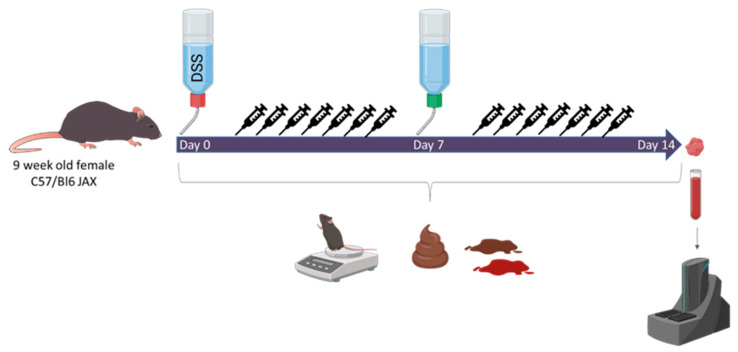
Overview of the study. Mice were treated with dextran sodium sulphate (DSS) during the first 7 days of the experiment and subsequently transferred back to regular drinking water. Daily disease activity was determined, and fecal pellets were scored and collected for microbiome analysis. At sacrifice, colonic tissue and blood were collected for RNA sequencing and serum cytokine analysis, respectively.

Study design: Nine-week-old female mice (*n* = 120) were allocated to 12 treatment groups (*n* = 10 per group) receiving either regular drinking water or 2.75% DSS. Mice were additionally treated daily until the end of the experiment at the same time through gavage (200 µL, 2 × 10^9^ CFU/mL) with PBS, Baker’s yeast (non-probiotic control), a non-acetate-producing Sb strain SDH1, the Sb Enterol^®^ strain ENT with transient acetate production but no accumulation (commercial probiotic control), a high-acetate-producing (5 g/L) Sb strain Sb.P, or a super-high-acetate-producing (8.5 g/L) Sb strain ENT3 (Table 1). Disease activity, including weight loss, diarrhea, and the presence of occult blood, was scored daily (Figure 1). On day 7, the DSS groups were transferred to regular drinking water and all mice were sacrificed on day 14. Blood was collected for cytokine and chemokine analysis using Mesoscale’s pro-inflammatory panel 1. Fecal pellets were collected in sterile Eppendorf’s with sterile forceps and subsequently snap frozen on dry ice to analyze microbial changes over time and conduct SCFA quantification. In addition, colonic tissue was collected at sacrifice and fixated in formaldehyde for histologic scoring and snap frozen for the evaluation of RNA expression levels by RNA sequencing.

Evaluation of inflammation: The Disease Activity Index (DAI) was determined daily by evaluating the body weight loss, stool consistency, and the presence of occult blood in the stools [24]. To assess macroscopical damage at sacrifice, the presence of adhesions, hyperemia, and the extent of colonic inflammation were assessed [25]. Histology of the colonic tissue was conducted on paraffin-embedded, 5 µm-thick longitudinal and transverse sections stained with hematoxylin and eosin. The microscopic inflammation score was calculated using previously established methods [25]. This score involved evaluating changes in mucosal architecture, neutrophil infiltration, mononuclear cell infiltration, goblet cell loss, and epithelial defects. An experienced pathologist (GdH) who was blinded for the experimental conditions evaluated the slides.

Blood serum cytokine and chemokine analysis: Cytokine levels were quantified in serum using the electro-chemi-luminescence-based Meso Scale Discovery (MSD) platform exploiting the V-PLEX Proinflammatory Panel 1 Mouse Kit. This kit provides assay-specific components for the quantitative determination of IFN-γ, IL-1β, IL-2, IL-4, IL-5, IL-6, IL-10, IL-12 p70, KC/GRO, and TNFα. Preparation of samples and detection plates were performed following the manufacturer’s instructions [Meso Scale Diagnostics, Rockville, MD, USA] with little adaptations. In short, a series of 9 concentrations of standards in duplicate, together with the samples in duplicate, were added to the plate. Plates were incubated while shaking at room temperature for 2 h. After incubation, plates were washed three times, and the detection antibody mixture was added to each well. Again, plates were incubated while shaking at room temperature for 2 h. Afterwards, plates were washed and a 2X Read buffer was added to each well. Plates were read on the MSD Plate reader [MESO QuickPlex SQ 120, Meso Scale Diagnostics, Rockville, MD, USA]. Protein concentrations were determined using the MSD Discovery Workbench 4.0 analysis software. Results are presented as absolute concentrations.

Short chain fatty acid measurements: Short chain fatty acid (SCFA) concentrations were measured in snap-frozen fecal samples collected on days 3 and 11 as previously described [26]. In short, fecal samples (100 mg) were suspended in 1 mL of saturated NaCl (36%) solution. An internal standard (50 μL of 10.7 µM 2-ethylbutyric acid in MQ water) was added and the samples were homogenized using glass beads. After the addition of 150 µL H_2_SO_4_ (96%), SCFAs were extracted with 3 mL of ether. The ether layer was collected and dried with Na_2_SO_4_ (150 mg). The supernatant (0.5 µL) was analyzed using gas chromatography with flame ionization detection (Agilent, Santa Clara, CA, USA). The system was equipped with a DB FFAP analytical column (30 m × 0.53 mm ID, 1.0 µm; Agilent, Santa Clara, PA, USA) and GC grade helium (5.6) was used as carrier gas with a constant flow of 4.2 mL/min. The initial oven temperature was held at 100 °C for 3 min, ramped at 4 °C/min to 140 °C (isothermal for 5 min) and then further ramped at 40 °C/min to 235 °C (isothermal for 15 min). The resulting chromatograms were processed using ChemStation (Agilent Technologies, Santa Clara, PA, USA).

Transcriptomic analysis: Snap-frozen colonic tissue underwent RNA extraction using the RNeasy mini-kit applying the manufacturer’s instructions [Qiagen, Germantown, MD, USA]. Subsequently, RNA libraries were prepared with the Lexogen quantseq kit [Lexogen, Vienna, Austria] and sequenced by Illumina HiSeq4000 [Illumina, San Diego, PA, USA], with an average sequencing depth of 15.97 million reads per sample. Alignment of raw RNA-sequencing data to the murine reference genome mm10 was performed through HISAT2; absolute counts were generated using HTSeq count. Post-normalization, differential expression analysis was performed using DESeq2 [27]. A false discovery rate (FDR) correction was applied, with a cut-off of 0.10. Biofunctional analysis and gene enrichment of significantly differentially expressed genes were performed with Ingenuity Pathway Analysis (IPA, Aarhus, Denmark).

Microbiome analysis: Snap-frozen fecal material collected at baseline, mid-experiment (day 9—highest clinical inflammation), and the end of the experiment (day 14), was submitted to DNA extraction and microbiota profiling as previously described [28]. In brief, DNA was extracted from fecal material using the MoBio PowerMicrobiome RNA isolation kit with the addition of 10 min incubation at 90 °C after the initial vortex step. The V4 region of the 16S rRNA gene was amplified with primer pair 515F/806R [28]. Sequencing was performed on the Illumina MiSeq platform (San Diego, CA, USA) to generate paired end reads of 250 bases in length in each direction. Fecal samples were processed, with modification of the protocol above, to dual-index barcoding, as described by Tito and colleagues [29]. After de-multiplexing using LotuS (version 1.565) [30], sequencing data pre-processing was performed using the DADA2 pipeline v1.6.0. [31], including trimming, quality control, merging of pairs, and taxonomic annotation using the GTDB classifier (r202) with default parameters.

Statistical analysis: Statistics were performed using GraphPad Prism 9 for clinical data and R version 4.3.0 for all sequencing data. A *p*- or *q*-value < 0.05 was considered statistically significant. Experimental *n* is reported throughout the methods and results, as well as in figure legends. Summary statistics are reported in the results section where appropriate. Graphs were generated using the ggpubr and ggplot2 packages in R version 4.3.0.

## 3. Results

### 3.1. High-Acetate-Producing Saccharomyces cerevisiae var. boulardii Strains Attenuate Inflammation and Show Dose-Dependent Histologic Improvement

**Figure 2:**
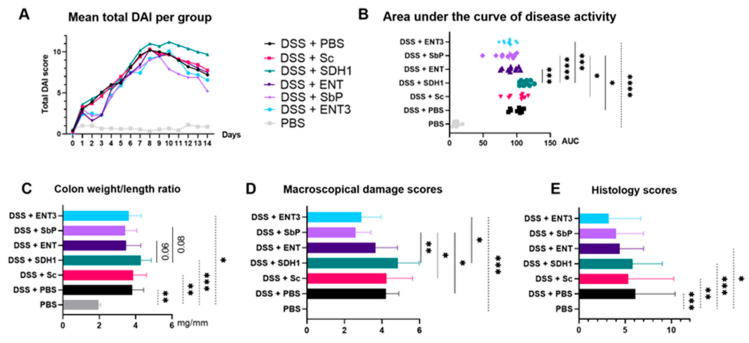
(**A**) Disease activity index (DAI) over time and (**B**) its evaluation by the area under the curve. Mean values per group are shown for DAI; individual values are shown per mice as well as the mean value for AUC. (**C**) Colon weight/length ratio (mean). (**D**) Macroscopical damage score (median). (**E**) Histology scores (mean). *p*-values (* *p* < 0.05; ** *p* < 0.01; *** *p* < 0.001, **** *p* < 0.0001) are given as the result of a one-way ANOVA followed by Dunn’s correction for parametric data and Kruskal–Wallis tests followed by Dunn’s correction for non-parametric data. Experimental *n* = 10.

Disease activity, determined by the area under the curve (Figure 2A,B), was significantly lower for strain Sb.P compared to the PBS control and Baker’s yeast (ANOVA followed by Dunn’s correction, *p* = 0.015 and *p* = 0.011, respectively). The SDH1 strain showed significantly higher disease activity compared to the Sb strains ENT (ANOVA followed by Dunn’s correction, *p* = 0.0009), Sb.P (*p* < 0.0001), and ENT3 (ANOVA followed by Dunn’s correction, *p* = 0.0001). At sacrifice, the colon weight/length ratio (Figure 2C) was decreased for the ENT and Sb.P strains compared to the SDH1 strain (Kruskal–Wallis tests followed by Dunn’s correction, *p* = 0.06 and *p* = 0.08, respectively). Moreover, macroscopical damage scores (Figure 2D) were significantly lower for Sb.P (Kruskal–Wallis tests followed by Dunn’s correction, *p* = 0.002) and ENT3 (Kruskal–Wallis tests followed by Dunn’s correction, *p* = 0.02) compared to SDH1. Additionally, these damage scores for Sb.P were significantly lower than the PBS control (Kruskal–Wallis tests followed by Dunn’s correction, *p* = 0.048) and Baker’s yeast (Kruskal–Wallis tests followed by Dunn’s correction, *p* = 0.033).

**Figure 3:**
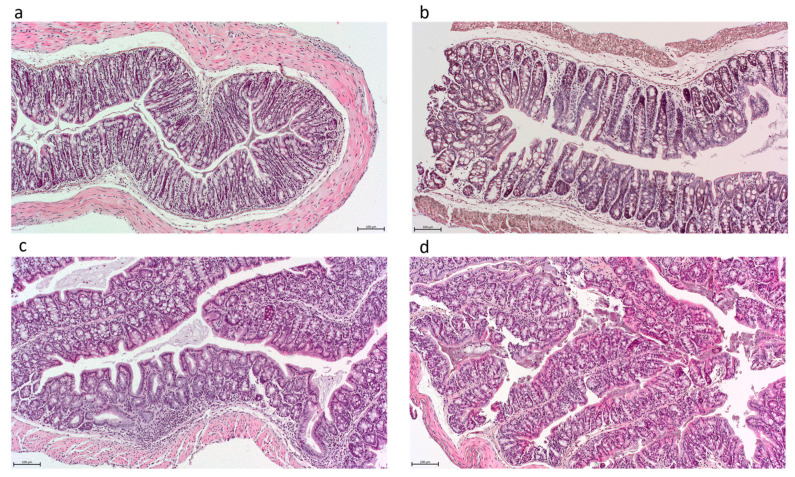
Histologic images of colonic tissue obtained independently from the received treatment with resp. scores: (**a**) score 0–normal tissue, (**b**) score 6, (**c**) score 11, (**d**) score 14. Scale bar = 100 µm.

Though not significant, a gradient was observed with regard to histologic inflamma-tion which was lowest upon administration of the highest-acetate-producing strain (Figure 2E and Figure 3). In comparison, significantly higher histologic inflammation was observed in the non- and transient-acetate-producing strains on DSS compared to the PBS controls (Kruskal–Wallis tests followed by Dunn’s correction, all *p* < 0.05), whereas this increase was not observed for both high-acetate-producing strains Sb.P and ENT3 on DSS (Figure 2E; *p* = NS)

### 3.2. Serum Cytokines Showed Mixed Profiles across Different Treatment Groups

**Figure 4:**
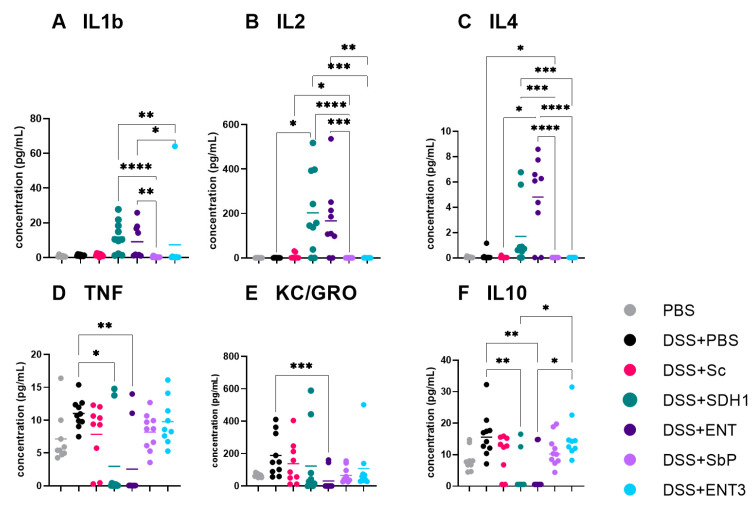
Serum cytokine levels of DSS-induced colitis groups evaluated by the mesoscale discovery platform. *p*-values (* *p* < 0.05; ** *p* < 0.01; *** *p* < 0.001, **** *p* < 0.0001) are given as the result of Kruskal–Wallis tests for non-parametric data. Experimental *n* = 10.

Different serum cytokine profiles were obtained between treatments. Significantly lower IL1β, IL2, and IL4 concentrations (Figure 4A–C) for Sb.P and ENT3, compared to SDH1 and ENT, were observed (Figure 4, Kruskal–Wallis tests followed by Dunn’s correction, all *p* < 0.05). In contrast, IL10, TNFα, and KC/GRO were significantly lower for the DSS groups on SDH1 and ENT compared to DSS groups on Sb.P and ENT3, and even to DSS + PBS for IL2 and IL4 (Figure 4D–F; Kruskal–Wallis tests followed by Dunn’s correction, all *p* < 0.05). Finally, compared to the PBS control, no differences (Kruskal–Wallis tests followed by Dunn’s correction, all *p* > 0.05) were observed between the different control groups for disease activity, colon weight/length ratio, and macroscopical damage score.

### 3.3. The Highest-Acetate-Producing Strain Exhibits Anti-Inflammatory Effects on Gene Expression

A significant effect of DSS-induced inflammation was observed at the level of RNA expression (Supplementary Figure S1, Kruskal–Wallis *p* < 0.05). Within the inflamed setting, some differential effects of treatment between ENT3 and other strains were observed at the RNA expression level (Table 2). Expression of *Tnf* was significantly lower for DSS + ENT3 compared to DSS + PBS (Wald test, *adj. p* < 0.1) and DSS + Sc (Wald test, *adj. p* < 0.1) and showed a trend that was not significant after multiple testing correction for DSS + SDH1 (Wald test, *p* = 0.05; *adj. p* = 0.42). No difference in *Tnf* expression was observed between ENT3 and both acetate-producing strains ENT and Sb.P (Wald test, all *p* > 0.10) upon inflammation. Additionally, *Il1b* was downregulated in DSS + ENT3 compared to DSS + PBS and DSS + Sc (Wald test, *adj. p* < 0.1), as well as DSS + SDH1 (Wald test, *p* < 0.01; *adj. p* = 0.18). Furthermore, some differences in barrier gene expression could be noted including *Cldn1, Cldn2*, and *Cldn8* (Table 2). Finally, evaluation of *S100a8* and *S100a9*, which are predominantly found as calprotectin, indicated (borderline) significant higher expression for DSS + PBS (Wald test, *adj. p* < 0.10*), DSS + Sc (*Wald test, *adj. p* < 0.10), and DSS + SDH1 (Wald test, *adj. p* = 0.18 and resp. *adj. p* = 0.43) and a numerical, yet non-significant upregulation for DSS + ENT (Wald test, Wald test, *unadj. p* = 0.11, *adj. p* = 0.88), compared to DSS + ENT3. Yet, no difference in *S100a8* and *S100a9* expression could be observed compared to DSS + Sb.P.

### 3.4. Induction of Colitis by DSS Administration Perturbs the Gut Microbiome 

**Figure 5:**
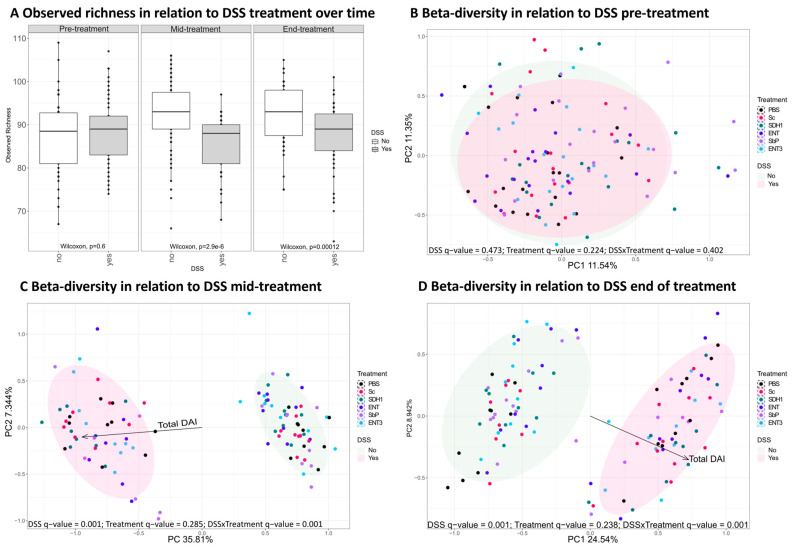
The effect of DSS inflammation on alpha-diversity defined by observed richness (**A**) and beta-diversity at the different timepoints pre (**B**), mid (**C**) and end (**D**) was independent of treatment over time. The given *q*-values are the *p*-values corrected for FDR. Experimental *n* = 10.

Upon induction of inflammation by DSS administration, a clear shift in alpha-diversity (Wilcoxon test, *p <* 0.05, Figure 5A) and beta-diversity (Adonis test, *q* = 0.001, Figure 5B–D) was observed.

At baseline, none of the identified bacterial taxa were differentially abundant for control groups versus those in which colitis was induced. Yet, upon induction of inflammation, several taxa responded with a change in their relative abundance compared to control mice, independently of the administered strain (Supplementary Table S1). Of note, significant decreases in members of the genera *Eubacterium*, *Lactobacillus*, and the unassigned genus *TF01-11* belonging to the *Lachnospiraceae* family, and significant increases in *Akkermansia* and *Faecalibaculum* compared to control mice (Wilcoxon test followed by BH correction, all *adj. p* < 0.05) were observed independently of timepoint during the study. Interestingly, *Acetatifactor* abundance was stable mid-experiment; however, it decreased at the end (Wilcoxon test followed by Benjamin-Hochberg, *p* = 9.13 × 10^−3^). Likewise, the abundance of *Emergencia* was initially stable but increased by the end of the experiment in the inflamed groups (Wilcoxon test followed by Benjamin-Hochberg, *p* = 1.24 × 10^−4^).

In line with the observed decreased abundance of butyrate-producing bacteria (e.g., *Eubacterium* spp. [32] and *TF01-11* [33]), shifts in fecal SCFA concentrations (Table 3), including reduced butyrate and increased acetate (mid-experiment only) and propionate concentrations, were observed upon induction of colitis.

### 3.5. Gut Microbial Species Richness Remains Consistently Preserved during Inflammation upon Treatment with the Highest-Acetate-Producing Saccharomyces cerevisiae var. boulardii Strains

**Figure 6:**
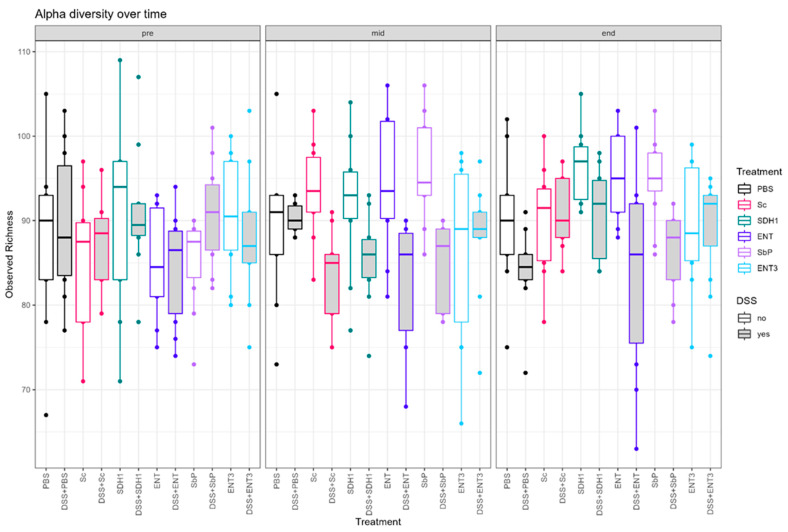
Trends in alpha-diversity defined by observed richness per condition over the track of the experiment. Experimental *n* = 10.

Differences in alpha-diversity were evaluated per treatment within inflamed and control subgroups (Figure 6). For the inflamed groups (Supplementary Figure S2), a difference between treatment groups mid-experiment could be observed (Kruskal–Wallis; *p* = 0.027). Subsequent, Dunn’s test identified only a significant difference between the DSS + PBS and DSS + Sc (all *p* < 0.05). At the end of the experiment, DSS + Sc, DSS + SDH1, and DSS + ENT3 show a trend for higher observed richness than DSS + PBS, but it is nonsignificant after multiple testing correction (uncorr. *p* = 0.005, *p* = 0.005 and *p* = 0.014, respectively; all *adj. p* > 0.05).

When evaluating alpha-diversity within treatment (Supplementary Table S4 and Supplementary Figure S3), no overall difference between inflamed and control groups could be observed for the high-acetate-producing ENT3 strain (Kruskal–Wallis *p* = 0.58, Supplementary Table S4), nor at any given timepoint separately (all *p* > 0.05, Table 4). This stability was not observed for any other treatment, although for the PBS control the difference was slightly delayed and for Sc the DSS effect is resolved at the last timepoint (Table 4).

Additionally, an effect of treatment could be observed on species composition. Beta-diversity was significantly different at these timepoints with, resp., *p* = 0.004 (mid) and *p* = 0.015 (end), and this was also reflected in the relative abundance of specific taxa (Supplementary Table S2). Mid-experiment, the abundance of *Alistipes* spp. was shown to be lower for all acetate-producing strains, ENT, Sb.P, and ENT3, compared to PBS, Sc, and SDH1 in the inflamed setting (Kruskal–Wallis followed by Dunn’s test, all *adj. p* < 0.05), whereas this was not the case at the end of the experiment. *Duncaniella* spp. showed decreased abundance in the PBS control, ENT, and ENT3 upon inflammation mid experimtent, but not at the end. At both the mid and end of the experiment, *Lactobacillus* spp. and *Limosilactobacillus* spp. were shown to be decreased upon treatment with SDH1 compared to the PBS control (Kruskal–Wallis followed by Dunn’s test, all *adj. p* < 0.05). Moreover, by the end of the experiment, more prominent differences in abundance upon treatment with SDH1 were observed (Supplementary Table S2). However, between the acetate-producing strains in the inflamed setting, limited specific differences were observed, e.g., the abundance of *Limosilactobacillus* spp. is higher for ENT compared to ENT3 (Supplementary Table S2). In addition, these findings were not associated with significant changes in SCFA concentrations upon treatment (Table 5).

In control mice (Supplementary Figure S4), no differences in observed richness were noted at any timepoint (all *p* > 0.05). However, an effect of treatment was observed in terms of beta-diversity, both at the mid and end of the experiment with *q* = 0.002 and *q* = 0.003, respectively (Adonis test). Hence, differential abundant genera were identified at the mid and end of the experiment (Supplementary Table S3).

## 4. Discussion

This preclinical study aimed to evaluate the effect of engineered high-acetate-producing *Saccharomyces cerevisiae* var. *boulardii* strains in an acute DSS model of colitis. A significant improvement in disease severity between mice receiving the Sb.P strain (high acetate production) and mice receiving PBS was demonstrated as expressed by the disease activity index. In comparison, administration of the original probiotic strain, marketed as Enterol^®^ and which has been demonstrated to have anti-inflammatory effects [34,35,36], displayed a quantitative yet non-significant difference in disease activity. Furthermore, a delayed disease activity was observed with acetate-producing strains, which might point to improved effects when applying a preventive treatment approach. Yet, this should be evaluated in future studies.

Various illness indicators were evaluated at sacrifice in addition to the clinical course of the disease. Of these, spleen weight roughly correlates with inflammatory response, although other factors such as dehydration or weight loss also have an effect, as previously demonstrated [37]; particularly the spleen weight of mice given DSS for four days increased significantly, whereas mice given DSS for seven days had a lighter spleen than control mice [37]. In the current study, there was no difference in spleen weight between the evaluated conditions. Furthermore, while spleen volume in humans has been linked to CD activity, this correlation was not confirmed in UC patients [38]. Although there were no significant variations in the colon weight/length ratio between the treatment groups at the sacrifice of the mice, differences between ENT and SDH1, as well as Sb.P and SDH1, were borderline significant. Furthermore, macroscopical inflammation scores differed significantly between Sb.P and SDH1 when compared to the PBS control. Taken together with the histology scores, these findings suggest that the presence of acetate-producing strains, and specifically Sb.P and ENT3, may alleviate inflammation and support recovery after DSS induced colitis.

Surprisingly, diseased mice receiving SDH1 showed a more severe disease course and a delayed recovery compared to the groups on baker′s yeast or PBS. However, this was not observed in the control mice receiving this strain. Considering that all the engineered yeasts in this study are genetically closely related, it would be highly interesting to pinpoint the specific genetic variations in follow-up studies and explore why these minor variations lead to more pronounced phenotypes in the presence of inflammation beyond the known mutations causing differences in acetate accumulation.

Different serum cytokine patterns were observed over the different conditions. Although the engineered yeast strains are genetically extremely close [23], they have different mutations causing their acetate accumulation [23]. The observed mixed results might indicate that serum cytokines are not fully exhibiting what is happening at the tissue level. Therefore, we also performed analysis of the pro-inflammatory gene expression in the colonic tissue. Subsequently, gene expression was shown to be impacted by inflammation as observed in previous studies [39]. Within the inflamed groups, similar trends for the clinical results were noted with decreased expression of *TNFα* in the highest-acetate-producing strain. Accordingly, compared to the highest-acetate-producing strain ENT3, *S100a8*, part of fecal calprotectin and serving as a biomarker for colitis, was shown to be significantly upregulated in the PBS and Sc controls, and there was a trend towards increased expression in the non-acetate-producing SDH1 strain and the ENT strain. Only minor differences could be observed for barrier-related genes and SCFA receptors. Surprisingly, the number of differentially expressed genes compared to DSS + ENT3 varied from high numbers for DSS + PBS, DSS + Sc, and DSS + Sb.P with, resp., 5010, 3009, and 1903 DEGs, to low numbers for DSS + ENT and SDH1 with, resp., 81 and 71 DEGs. This could be caused by the different genetic backgrounds and subsequent effects, as both SDH1 and ENT3 were engineered from the parent ENT strain and Sb.P is the result of a natural mutation.

Since it is well documented that the microbiome plays a major role in the pathogenesis of IBD [4,40], microbial analysis was performed to evaluate the effects of these strains on the microbiota composition of the different strains. Upon induction of inflammation, a clear shift in alpha- and beta-diversity was observed, confirming previous findings [36]. However, for ENT3 the highest-acetate-producing strain, within-treatment analysis could not identify any difference in observed richness over the track of the experiment. Moreover, paired analysis indicated that in inflamed settings ENT3 could indeed prevent a decrease in alpha-diversity upon induction of inflammation over the whole track of the experiment. Contradictory, paired analysis with healthy controls showed an increase for the acetate-producing strains ENT (transient) and Sb.P (high), but not for ENT3 (extra-high), where alpha-diversity remained stable. Beta-diversity was shown to be affected by the received treatment, and subsequent analyses at the taxa level indicated differentially abundant bacterial strains. An increase in *Alistipes* spp. and *Duncaniella* spp. have recently been associated with disease severity in DSS mice models [41]. Here, *Alistipes* spp. were decreased for all acetate-producing strains upon inflammation. *Duncaniella* spp. on the other hand showed reduced abundance in the PBS control, and ENT and ENT3 in the inflamed setting. Taken together, these findings might point towards beneficial effects of the respective treatments. The beneficial [42,43] *Lactobacillus* spp. and *Limosilactobacillus* spp. were shown to be decreased in SDH1 treated mice upon inflammation, allowing us to speculate that this might have contributed to the disease severity. Conversely, in controls, the abundance of both taxa was increased upon administration of ENT3, potentially explaining its effects. Additionally, in controls, *Muribaculaceae,* previously negatively correlated with DSS-colitis, were shown to be increased for all acetate-producing strains, thus suggesting beneficial effects [44]. While these findings support further research on the potential application of high-acetate-producing strains in a preventive context, microbial extrapolation from a mice model to the human setting is not straightforward and should be performed carefully. However, we here showed that there might be a role for (engineered) acetate-producing *Saccharomyces* strains to maintain a healthy gut microbiota composition.

Similar concentrations of acetate were expected to be produced by the Sb.P strain, and even higher concentrations by the ENT3 strain, in comparison to those used in our in vitro studies [45]. Yet, high doses of acetate may lower the gut environmental pH too drastically or reach a physiological upper-limit upon which all the accumulated acetate cannot be consumed by bacteria or intestinal epithelial cells. On the other hand, probiotic delivery of acetate might be safer compared to direct post-biotic [46] delivery of the chemical, as it allows the yeast to attach to the mucosa and locally and gradually accumulate acetate. Moreover, this approach might be preferred by IBD patients as this method of delivery requires less frequent intake.

Although the ENT3 strain, which accumulates the highest quantities of acetate in vitro, was not associated with improved disease severity based on the macroscopical scoring, a dose-response was observed at the histologic level with the lowest histologic inflammation obtained with ENT3 followed by Sb.P and ENT, followed by both controls (PBS and Sc), and finally, the SDH1 strain which lacks acetate accumulation, showed the highest histologic inflammation score. While one could speculate these observations might be correlated with differences in acetate production capacity of the administered strains, this was not mirrored by the fecal concentrations of acetate or other SCFAs measured in the inflamed setting. However, due to bacterial and host-level metabolization of SCFAs, as well as additional production by autochthonous gut microbiota, interpretation of these findings should be performed carefully. In the future, it would be interesting to explore if the acetate or other SCFAs produced by engineered probiotic strains could be labelled in order to fully evaluate the origin of the measured concentrations.

Limitations of this work are related to extrapolation of results from the in vivo mice model to the human situation. DSS-induced colitis has some similarities to UC, as it also increases intestinal barrier permeability in mice and causes weight loss. However, the complexity of UC cannot be fully replicated in this mouse model. In terms of microbiota evaluation, implementing humanization of the mice’s gut microbiota [35] should be considered in further work. Nevertheless, this mice model adds other layers of complexity compared to in vitro methods in which co-culturing with immune cells and microbiota is not yet fully established [45].

## 5. Conclusions

In conclusion, engineered extra-high-acetate-producing *Saccharomyces cerevisiae* var. *boulardii* strains showed indications based on histology and transcriptomic expression levels towards attenuation of DSS-induced colitis compared to the parent *Saccharomyces cerevisiae* var. *boulardii* strain. A clearer effect on retaining gut microbial richness during inflammation was observed. Unexpectedly, the engineered *Saccharomyces cerevisiae* var. *boulardii* strain that did not produce acetate at all, increased inflammation. Furthermore, control mice did not show clinical side effects indicating the strains to be safe. Together with our previous in vitro work [45] exploiting a patient-derived human epithelial cell culture model which showed a protective effect of high acetate administration on epithelial permeability, gene expression, and cytokine production, these findings support further research on the possibilities for acetate-mediated management of barrier defects and inflammation in IBD by high-acetate-producing *Saccharomyces cerevisiae* var. *boulardii* strains.

## 6. Patents

VIB and KU Leuven have submitted patent applications (15 September 2017; EP 17191252.0. and 27 January 2022; EP 22153700.4) based on the possible beneficial effect of *S. boulardii* produced acetic acid for its commercial use as probiotic. This work has resulted in an international patent application entitled ‘Improved probiotic potency of the yeast Saccharomyces boulardii’ [PCT/EP2023/051941].

## Figures and Tables

**Table 1:** Overview of the different oral treatments including controls and Saccharomyces strains.

Strain	Selection Criterion	Acetate Accumulation
PBS	Negative control	0 g/L
*S. cerevisiae* [Sc]	Non-probiotic control	0 g/L
*S. cerevisiae* var. *boulardii* SDH1 [SDH1]	No acetate production (engineered)	0 g/L
*S. cerevisiae* var. *boulardii* Enterol [ENT]	Probiotic control	Transient with 1.8 g/L peak at 24 h, but 0 g/L after 48 h
*S. cerevisiae* var. *boulardii* Sb.P [Sb.P]	High acetate accumulation (natural mutation)	5 g/L
*S. cerevisiae* var. *boulardii* ENT3 [ENT3]	Highest acetate accumulation (engineered)	8.5 g/L

**Table 2:** List of selected genes of interest and their fold changes (log2fold, given by FC) for the given condition compared to DSS + ENT3 (the highest acetate producer) and their corresponding adjusted *p* value. (*) Significant prior to transcriptome-wide multiple testing correction. Experimental *n* = 10, NA = not available.

	Gene	DSS + PBS		DSS + Sc		DSS + SDH1		DSS + ENT		DSS + Sb.P	
		FC	*Adj. p*	FC	*Adj. p*	FC	*Adj. p*	FC	*Adj. p*	FC	*Adj. p*
Inflammatory genes	*Tnf*	1.31	<0.01	1.55	<0.01	0.88	0.42 *	0.48	0.74	−0.29	NA
	*Il1b*	2.49	<0.01	4.6	<0.01	1.77	0.18 *	0.42	0.84	0.29	NA
	*Il2*	−0.55	0.36	−0.74	0.26	−0.71	0.58	0.25	0.86	−0.65	NA
	*Il12a*	1.62	<0.01	0.89	0.22 *	0.005	0.99	0.24	0.89	0.01	NA
Barrier genes	*Muc2*	−0.02	0.97	0.07	0.88	−0.008	0.99	0.03	0.98	0.04	0.94
	*Cldn1*	1	0.03	0.75	0.16 *	0.1	0.94	0.77	0.49	0.44	NA
	*Cldn2*	−0.25	0.43	−0.4	0.22 *	−0.54	0.33	−0.5	0.44	−0.76	0.04
	*Cldn8*	1.38	<0.01	1.3	<0.01	0.64	0.25	0.05	0.95	−0.15	0.68
	*Tjp1~ZO1*	0.13	0.63	0.34	0.2	0.31	0.54	−0.07	0.91	0.56	0.06
	*Ocel1~OCLDN*	0.07	0.75	−0.09	0.73	0.16	0.71	0.3	0.5	0.06	0.79
CAMs	*Icam1*	1.29	<0.01	1	<0.01	0.55	0.47	0.28	0.76	0.14	0.75
Proliferation marker	*Mki67*	0.21	0.49	0.47	0.14 *	0.09	0.91	0.2	0.8	0.07	0.86
SCFA receptors	*Ffar2*	0.26	0.37	0.26	0.43	0.37	0.54	0.1	0.89	−0.13	0.72
	*Ffar3*	0.32	0.33	−0.08	0.85	−0.06	0.94	0.21	0.81	0.13	0.76
	*Ffar4*	0.16	0.67	−0.1	0.81	−0.33	0.65	0.14	0.87	−0.64	0.1
	*Hcar2*	0.46	0.3	1.02	0.02	−0.12	0.92	0.24	0.84	0.22	NA
Model related genes	*S100a8*	4	<0.01	5.57	<0.01	2.67	0.18 *	1.36	0.66 *	0.88	NA
	*S100a9*	3.55	<0.01	5.1	<0.01	1.99	0.43 *	0.68	0.83	0.88	NA
	*Lcn2*	0.77	0.23	1.71	0.01	0.46	0.76	0.03	0.99	−0.65	NA

**Table 3:** Fecal SCFA concentrations (mean [SD]) at the mid and end of the experiment of controls and DSS-induced colitis mice (each *n* = 60).

	Mid-Experiment			End of the Experiment		
SCFA	Controls	DSS Colitis	*p (t*-Test)	Controls	DSS Colitis	*p (t*-Test)
Acetate	59.36 [24.63]	75.28 [22.95]	<0.001	74.78 [20.25]	78.25 [23.90]	0.399
Propionate	4.60 [2.10]	6.32 [1.92]	<0.001	6.90 [2.46]	9.86 [4.02]	<0.001
Butyrate	6.64 [5.64]	3.18 [1.61]	<0.001	9.36 [5.22]	6.51 [5.20]	0.004

**Table 4:** Results of Wilcoxon test comparing the alpha-diversity defined by observed richness of the inflamed condition with their respective healthy controls receiving the same treatment at the different timepoints.

Comparison	Pre (*p*)	Mid (*p*)	End (*p*)
PBS vs. DSS + PBS	1.00	1.00	0.049
Sc vs. DSS + Sc	0.47	0.0003	0.71
SDH1 vs. DSS + SDH1	0.77	0.037	0.04
ENT vs. DSS + ENT	0.85	0.015	0.037
Sb.P vs. DSS + Sb.P	0.08	0.005	0.0048
ENT3 vs. DSS + ENT3	0.41	0.93	0.8

**Table 5:** Fecal SCFA concentrations in mM (Mean [SD)] in the different inflamed conditions with *p*-values being the result of an ANOVA. Experimental *n* = 10.

	Acetate		Propionate		Butyrate	
Group	Mid	End	Mid	End	Mid	End
DSS + PBS	77.19 [19.4]	89.06 [38.7]	6.78 [1.50]	10.05 [5.24]	3.84 [1.82]	7.30 [6.65]
DSS + Sc	78.65 [23.7]	80.44 [16.1]	6.84 [1.33]	9.97 [3.99]	3.66 [1.81]	5.41 [1.83]
DSS + SDH1	76.03 [18.8]	80.57 [22.0]	6.37 [1.45]	10.94 [2.89]	2.60 [0.82]	8.81 [8.00]
DSS + Sb.P	67.45 [32.0]	76.16 [17.1]	5.87 [2.79]	9.54 [3.98]	3.49 [1.93]	5.47 [2.95]
DSS + ENT	73.93 [20.0]	72.16 [23.4]	6.12 [2.29]	9.79 [5.46]	2.60 [1.40]	6.15 [5.73]
DSS + ENT3	78.39 [25.3]	70.72 [19.4]	5.95 [2.00]	8.88 [2.71]	2.88 [1.56]	5.77 [3.53]
*p*	0.9	0.58	0.81	0.93	0.33	0.68

## Data Availability

The data presented in this study are available on request from the corresponding author. The data are not publicly available due to legal reasons related to IP.

## Supplementary Materials

The following supporting information can be downloaded at: https://www.mdpi.com/article/10.3390/nu16162668/s1, Table S1: Significant differential abundant bacteria upon inflammation compared to controls at the mid and end of the experiment, Table S2: Significant differential abundant bacteria within inflamed groups the mid and end of the experiment, Table S3: Significant differential abundant bacteria within healthy controls at the mid and end of the experiment, Table S4: Results of Wilcoxon test comparing the alpha-diversity defined by observed richness of the inflamed condition with their respective healthy controls receiving the same treatment at the different timepoints, Figure S1: PCA-plots for transcriptomic analysis of colon tissue (A) effect of inflammation, (B) cage-effect, and (C) effect of condition (inflammation and treatment), Figure S2: Observed richness (alpha-diversity) in inflamed subgroups at the different timepoints. (A) Baseline (B) Mid-experiment (C) End of the experiment, Figure S3: Observed richness (alpha-diversity) within treatment and timepoint, Figure S4: Observed richness (alpha-diversity) in healthy subgroups at the different timepoints. (A) Baseline (B) Mid-experiment (C) End of the experiment.

## Abbreviations

CD	Crohn’s Disease
DSS	Dextran sodium sulphate
ENT	Enterol-Saccharomyces cerevisiae var. boulardii strain
ENT3	Extra-high-acetate-producing *Saccharomyces cerevisiae* var. *boulardii* strain
IBD	Inflammatory bowel disease
PBS	Phosphate buffered saline
Sb	Saccharomyces cerevisiae var. boulardii
Sc	Saccharomyces cerevisiae
Sb.P	High-acetate-producing *Saccharomyces cerevisiae* var. *boulardii* strain
SCFA	Short chain fatty acids
SDH1	Non-acetate-producing *Saccharomyces cerevisiae* var. *boulardii* strain
UC	Ulcerative colitis
