# Supplementary material for: Effect of Mutant and Engineered High-Acetate-Producing Saccharomyces cerevisiae var. boulardii Strains in Dextran Sodium Sulphate-Induced Colitis
Source: Nutrients. 2024 Aug 13;16(16):2668. doi: 10.3390/nu16162668 (PMC11357622; doi:10.3390/nu16162668)

1 Supplementary material

1.1 Supplementary tables

Supplementary Table S1. Significant differential abundant bacteria upon inflammation compared to controls at mid and end experiment.

Dir. Up (more) or down (less) abundant than in the PBS control condition. P-values are the results of Wilcoxon-tests followed by the Benjamini-Hochberg procedure for multiple comparison correction (adj.p).

**Taxa**	**Mid**			**End**		
	**Dir.**	***p***	***Adj. p***	**Dir.**	***p***	***Adj. p***
g_14-2	down	5.26E-14	3.54E-13	down	2.57E-12	2.80E-11
g_Acetatifactor	=	ns	ns	down	4.26E-03	9.13E-03
g_Acutalibacter	down	2.44E-02	3.64E-02	down	3.53E-05	1.01E-04
g_Adlercreutzia	down	6.79E-06	1.75E-05	down	3.63E-09	2.56E-08
g_Akkermansia	up	5.00E-21	2.02E-19	up	3.37E-18	9.93E-17
g_Alistipes	up	5.91E-06	1.59E-05	up	6.00E-06	2.06E-05
g_AM07-15	down	3.99E-07	1.27E-06	down	2.20E-06	7.75E-06
g_Anaerofustis	down	3.91E-06	1.10E-05	=	ns	ns
g_Anaerosacchariphilus	down	8.81E-05	1.87E-04	=	ns	ns
g_Anaerosporobacter	up	3.23E-04	6.11E-04	=	ns	ns
g_Anaerotruncus	down	1.64E-06	4.96E-06	=	ns	ns
g_Angelakisella	down	2.97E-04	5.70E-04	down	7.08E-07	2.93E-06
g_Bacteroides	up	1.47E-16	1.78E-15	up	2.31E-11	1.98E-10
g_C-19	down	2.28E-14	1.72E-13	down	1.13E-09	8.49E-09
g_C-53	up	2.15E-05	5.00E-05	=	ns	ns
g_CAG-115	up	2.17E-04	4.30E-04	down	2.12E-02	3.64E-02
g_CAG-475	up	2.72E-05	6.22E-05	=	ns	ns
g_CAG-485	down	3.70E-08	1.44E-07	down	1.05E-06	4.07E-06
g_CAG-495	up	7.30E-03	1.13E-02	=	ns	ns
g_CAG-56	down	1.21E-16	1.63E-15	down	1.92E-14	3.29E-13
g_CAG-878	=	ns	ns	up	1.92E-02	3.34E-02
g_CAG-95	down	5.06E-04	9.41E-04	=	ns	ns
g_Caprobacter	down	2.53E-04	4.93E-04	down	1.90E-02	3.34E-02
g_CHKCI006	=	ns	ns	up	8.53E-07	3.41E-06
g_COE1	=	ns	ns	down	5.28E-04	1.38E-03
g_Clostridium	up	7.48E-21	2.26E-19	up	1.13E-11	1.04E-10
g_D16-34	down	7.95E-06	2.00E-05	down	6.17E-03	1.24E-02
g_DNF00809	down	1.54E-07	5.48E-07	down	1.01E-05	3.18E-05
g_Duncaniella	down	1.54E-12	8.88E-12	down	1.11E-13	1.66E-12
g_Dwaynesavagella	down	5.40E-03	8.70E-03	down	ns	ns
g_Emergencia	=	ns	ns	up	6.28E-03	1.24E-02
g_Enterocloster	down	2.73E-02	3.98E-02	down	1.38E-02	2.56E-02
g_Erysipelatoclostridium	up	6.59E-17	9.97E-16	up	4.14E-18	9.93E-17
g_Eubacterium_F	down	4.14E-17	7.16E-16	down	3.09E-16	6.17E-15
g_Eubacterium_J	down	3.49E-16	3.84E-15	down	1.09E-18	4.37E-17

g_Evtepia	down	8.12E-06	2.00E-05	down	ns	ns
g_Faecalibaculum	**up**	1.05E-04	2.12E-04	**down**	1.24E-06	4.49E-06
g_Flavonifractor	down	1.68E-02	2.58E-02	down	9.15E-04	2.20E-03
g_Hungatella_A	down	1.75E-02	2.64E-02	down	5.88E-04	1.50E-03
g_Intestinibacillus	up	6.74E-04	1.24E-03	up	7.58E-04	1.89E-03
g_Kineothrix	=	ns	ns	down	1.46E-03	3.32E-03
g_Lachnoclostridium_A	down	1.54E-07	5.48E-07	down	1.04E-03	2.45E-03
g_Lactobacillus	down	5.00E-16	5.04E-15	down	6.67E-03	1.27E-02
g_Ligilactobacillus	up	1.41E-10	6.82E-10	=	ns	ns
g_Limosilactobacillus	down	2.73E-14	1.95E-13	=	ns	ns
g_MD308	down	7.20E-11	3.96E-10	down	4.43E-08	2.53E-07
g_Merdimonas	down	1.02E-10	5.36E-10	down	6.91E-08	3.61E-07
g_Monoglobus	down	2.86E-07	9.60E-07	=	ns	ns
g_Mucispirillum	up	1.67E-05	3.96E-05	=	ns	ns
g_Murimonas	down	3.84E-21	2.02E-19	down	3.37E-19	2.02E-17
g_Odoribacter	down	1.96E-03	3.38E-03	down	7.78E-08	3.89E-07
g_Parabacteroides	up	5.78E-10	2.69E-09	up	2.43E-04	6.64E-04
g_Paramuribaculum	down	1.18E-13	7.49E-13	down	3.17E-12	3.17E-11
g_Parasutterella	down	3.14E-03	5.24E-03	=	ns	ns
g_Prevotella	down	9.04E-04	1.63E-03	=	ns	ns
g_Prevotellamassilia	down	6.28E-09	2.62E-08	=	ns	ns
g_Pseudocitrobacter	up	5.66E-18	1.14E-16	up	1.44E-13	1.92E-12
g_Romboutsia	up	2.19E-18	5.31E-17	up	4.96E-09	3.13E-08
g_RUG13077	up	2.41E-06	6.94E-06	=	ns	ns
g_RUG13160	up	5.93E-03	9.43E-03	up	4.07E-04	1.08E-03
g_RUG350	up	1.76E-08	7.10E-08	up	7.89E-06	2.63E-05
g_Ruminococcus_F	up	1.25E-13	7.57E-13	up	2.31E-07	1.07E-06
g_Ruthenibacterium	up	4.26E-06	1.17E-05	up	6.35E-08	3.47E-07
g_Schaedlerella	up	9.72E-05	1.99E-04	up	4.41E-03	9.28E-03
g_Senegalimassilia	down	6.94E-03	1.09E-02	=	ns	ns
g_Soleaferrea	down	2.19E-07	7.56E-07	down	8.77E-08	4.21E-07
g_Streptococcus	up	2.99E-15	2.41E-14	up	2.73E-07	1.22E-06
g_TF01-11	down	1.10E-21	1.33E-19	down	9.19E-22	1.10E-19
g_Turicibacter	up	1.93E-15	1.66E-14	up	1.22E-05	3.75E-05
g_UBA1417	up	3.06E-05	6.85E-05	up	1.08E-03	2.48E-03
g_UBA3402	down	6.44E-08	2.43E-07	down	3.22E-05	9.42E-05
g_UBA6985	up	2.63E-02	3.88E-02	up	1.62E-02	2.94E-02
g_UBA9502	down	1.70E-09	7.33E-09	down	2.73E-02	4.55E-02
g_UMGS1864	up	3.96E-03	6.48E-03	up	1.51E-04	4.21E-04
g_UMGS1872	down	5.36E-05	1.18E-04	down	2.04E-08	1.23E-07
uc_c_Clostridia	=	ns	ns	up	1.50E-03	3.32E-03
uc_f_Acutalibacteraceae	down	1.27E-03	2.26E-03	down	6.21E-03	1.24E-02
uc_f_Anaerovoracaceae	down	6.37E-10	2.86E-09	down	9.03E-06	2.93E-05
uc_f_Atopobiaceae	down	3.56E-07	1.16E-06	down	2.60E-13	3.12E-12
uc_f_Bacteroidaceae	up	2.17E-06	6.41E-06	up	4.10E-10	3.28E-09
uc_f_Desulfovibrionaceae	down	3.16E-03	5.24E-03	down	6.57E-03	1.27E-02
uc_f_Eggerthellaceae	down	1.40E-05	3.39E-05	down	1.14E-06	4.29E-06
uc_f_Gastranaerophilaceae	up	8.16E-16	7.60E-15	up	4.30E-09	2.87E-08

uc_f_Muribaculaceae	up	9.49E-05	1.98E-04	=	ns	ns
uc_f_Oscillospiraceae	down	2.71E-03	4.61E-03	=	ns	ns
uc_f_UBA660	=	ns	ns	up	1.70E-02	3.05E-02
uc_k_Bacteria	down	1.68E-03	2.94E-03	down	2.51E-02	4.24E-02
uc_o_Bacteroidales	up	1.16E-10	5.85E-10	=	ns	ns
uc_o_Christensenellales	up	6.79E-06	1.75E-05	up	2.31E-03	5.05E-03
uc_o_Lachnospirales	down	1.24E-06	3.85E-06	down	4.84E-03	1.00E-02
uc_p_Firmicutes_A	up	8.40E-05	1.81E-04	up	1.33E-02	2.49E-02

Supplementary Table S2. Significant differential abundant bacteria within inflamed groups mid and end experiment.

P-values are the results of Kruskal-Wallis tests which were followed by the Dunn's correction for multiple comparison (adj.p). Direction of changes is given by the Z-values (positive values are for higher abundance, whereas negative values are for lower abundance).

Taxa mid	chi2	z	p	Adj.p	Comparisons
g_Alistipes	22.69	-2.80	0.00	0.01	DSS+ENT - DSS+PBS
g_Alistipes	22.69	-2.80	0.00	0.01	DSS+ENT - DSS+Sc
g_Alistipes	22.69	-2.65	0.00	0.01	DSS+ENT - DSS+SDH1
g_Alistipes	22.69	-3.04	0.00	0.02	DSS+ENT3 - DSS+PBS
g_Alistipes	22.69	-3.03	0.00	0.01	DSS+ENT3 - DSS+Sc
g_Alistipes	22.69	-2.88	0.00	0.01	DSS+ENT3 - DSS+SDH1
g_Alistipes	22.69	2.49	0.01	0.01	DSS+PBS - DSS+Sb.P
g_Alistipes	22.69	-2.50	0.01	0.01	DSS+Sb.P - DSS+Sc
g_Alistipes	22.69	-2.34	0.01	0.02	DSS+Sb.P - DSS+SDH1
g_CAG-1031	12.92	2.33	0.01	0.05	DSS+ENT3 - DSS+PBS
g_CAG-1031	12.92	3.14	0.00	0.01	DSS+ENT3 - DSS+Sb.P
g_CAG-485	10.47	-2.46	0.01	0.03	DSS+ENT3 - DSS+Sc
g_CAG-485	10.47	-2.49	0.01	0.05	DSS+PBS - DSS+Sc
g_Duncaniella	20.05	-2.68	0.00	0.02	DSS+ENT - DSS+Sc
g_Duncaniella	20.05	-3.83	0.00	0.00	DSS+ENT3 - DSS+Sc
g_Duncaniella	20.05	-3.79	0.00	0.00	DSS+PBS - DSS+Sc
g_Lactobacillus	9.60	2.78	0.00	0.04	DSS+PBS - DSS+SDH1
g_Limosilactobacillus	11.30	2.72	0.00	0.05	DSS+PBS - DSS+SDH1
g_Turicibacter	9.43	-2.92	0.00	0.03	DSS+Sb.P - DSS+SDH1
uc_f_Muribaculaceae	16.07	-2.78	0.00	0.01	DSS+PBS - DSS+Sc
uc_f_Muribaculaceae	16.07	-2.45	0.01	0.03	DSS+PBS - DSS+SDH1
uc_f_Muribaculaceae	16.07	-3.13	0.00	0.01	DSS+Sb.P - DSS+Sc
uc_f_Muribaculaceae	16.07	-2.83	0.00	0.02	DSS+Sb.P - DSS+SDH1
**Taxa end**	**chi2**	**z**	**p**	**Adj.p**	**Comparisons**
g_Lactobacillus	11.11	2.85	0.00	0.03	DSS+ENT - DSS+SDH1
g_Lactobacillus	11.11	2.77	0.00	0.02	DSS+Sb.P - DSS+SDH1
g_Prevotellamassilia	10.42	2.66	0.00	0.03	DSS+ENT - DSS+SDH1
g_COE1	10.05	2.84	0.00	0.03	DSS+ENT3 - DSS+Sc
g_Limosilactobacillus	14.72	2.37	0.01	0.03	DSS+ENT - DSS+ENT3
g_Limosilactobacillus	14.72	2.51	0.01	0.03	DSS+ENT - DSS+PBS
g_Limosilactobacillus	14.72	2.97	0.00	0.02	DSS+ENT - DSS+SDH1
g_Limosilactobacillus	14.72	2.56	0.01	0.04	DSS+Sb.P - DSS+SDH1
g_Paramuribaculum	12.13	2.60	0.00	0.03	DSS+ENT - DSS+SDH1

g_Paramuribaculum	12.13	3.07	0.00	0.02	DSS+PBS - DSS+SDH1
g_Faecalibaculum	8.41	-2.87	0.00	0.03	DSS+PBS - DSS+Sb.P
g_UMGS268	17.38	-2.86	0.00	0.02	DSS+ENT - DSS+Sc
g_UMGS268	17.38	-2.61	0.00	0.02	DSS+ENT3 - DSS+Sc
g_UMGS268	17.38	-3.02	0.00	0.02	DSS+Sb.P - DSS+Sc
g_UMGS268	17.38	-2.54	0.01	0.02	DSS+ENT - DSS+SDH1
g_UMGS268	17.38	-2.28	0.01	0.03	DSS+ENT3 - DSS+SDH1
g_UMGS268	17.38	-2.71	0.00	0.02	DSS+Sb.P - DSS+SDH1
g_Prevotella	24.86	-2.71	0.00	0.01	DSS+ENT - DSS+PBS
g_Prevotella	24.86	2.96	0.00	0.01	DSS+ENT3 - DSS+Sb.P
g_Prevotella	24.86	4.52	0.00	0.00	DSS+PBS - DSS+Sb.P
g_Prevotella	24.86	3.25	0.00	0.00	DSS+PBS - DSS+Sc
g_Prevotella	24.86	3.29	0.00	0.00	DSS+PBS - DSS+SDH1
uc_f_Gastranaerophilaceae	11.20	-3.11	0.00	0.01	DSS+ENT3 - DSS+SDH1
g_Kineothrix	14.47	-2.52	0.01	0.03	DSS+ENT3 - DSS+Sb.P
g_Kineothrix	14.47	-3.05	0.00	0.02	DSS+ENT3 - DSS+SDH1
g_Kineothrix	14.47	-2.67	0.00	0.03	DSS+Sc - DSS+SDH1
g_Schaedlerella	12.17	-3.34	0.00	0.01	DSS+ENT3 - DSS+Sc

Supplementary Table S3. Significant differential abundant bacteria within healthy controls mid and end experiment.

P-values are the results of Kruskal-Wallis tests which were followed by the Dunn's correction for multiple comparison (adj.p). Direction of changes is given by the Z-values (positive values are for higher abundance, whereas negative values are for lower abundance).

**Taxa mid**	**chi2**	**z**	***p***	***Adj.p***	**Comparisons**
g_Anaerosacchriphilus	14.49	-3.09	0.00	0.01	ENT3 - Sb.P
g_Anaerosacchriphilus	14.49	-2.47	0.01	0.03	PBS - Sb.P
g_Anaerosacchriphilus	14.49	3.09	0.00	0.02	Sb.P - SDH1
g_Anaerosporobacter	13.61	2.84	0.00	0.02	ENT3 - SDH1
g_Anaerosporobacter	13.61	2.77	0.00	0.01	PBS - SDH1
g_Anaerosporobacter	13.61	2.43	0.01	0.03	Sb.P - SDH1
g_Anaerosporobacter	13.61	3.03	0.00	0.02	Sc - SDH1
g_Anaerotignum	12.64	2.77	0.00	0.02	ENT3 - PBS
g_Anaerotignum	12.64	-2.39	0.01	0.04	PBS - Sc
g_Anaerotignum	12.64	-3.15	0.00	0.01	PBS - SDH1
g_Clostridium	15.52	3.03	0.00	0.01	ENT - ENT3
g_Clostridium	15.52	2.95	0.00	0.01	ENT - PBS
g_Clostridium	15.52	3.03	0.00	0.01	ENT - Sb.P
g_Clostridium	15.52	3.03	0.00	0.02	ENT - Sc
g_DNF00809	18.16	2.83	0.00	0.01	ENT - ENT3
g_DNF00809	18.16	2.84	0.00	0.01	ENT - PBS
g_DNF00809	18.16	3.74	0.00	0.00	ENT - Sb.P
g_DNF00809	18.16	3.43	0.00	0.00	ENT - Sc
g_DNF00809	18.16	3.05	0.00	0.01	ENT - SDH1
g_DUPI01	8.90	-2.83	0.00	0.03	PBS - Sb.P
g_Dwaynesavagella	19.12	-2.76	0.00	0.01	ENT - Sb.P
g_Dwaynesavagella	19.12	-3.09	0.00	0.01	ENT - SDH1
g_Dwaynesavagella	19.12	-2.29	0.01	0.03	ENT3 - Sb.P
g_Dwaynesavagella	19.12	-2.63	0.00	0.01	ENT3 - SDH1
g_Dwaynesavagella	19.12	2.76	0.00	0.01	Sb.P - Sc
g_Dwaynesavagella	19.12	-3.09	0.00	0.01	Sc - SDH1
g_Erysipelatoclostridium	10.55	2.41	0.01	0.04	ENT - ENT3

g_Hungatella_A	9.57	-2.75	0.00	0.05	Sb.P - SDH1
g_Hydrogenoanaerobacterium	10.01	-2.68	0.00	0.03	ENT3 - Sc
g_Ligilactobacillus	9.94	2.98	0.00	0.02	PBS - SDH1
g_Muribaculum	14.33	2.28	0.01	0.03	ENT - PBS
g_Muribaculum	14.33	2.37	0.01	0.03	ENT - Sc
g_Muribaculum	14.33	2.43	0.01	0.03	ENT3 - PBS
g_Muribaculum	14.33	2.53	0.01	0.04	ENT3 - Sc
g_Muribaculum	14.33	-2.45	0.01	0.04	PBS - SDH1
g_NSJ-38	11.72	-2.51	0.01	0.05	ENT - PBS
g_NSJ-38	11.72	-2.90	0.00	0.03	ENT - Sb.P
g_Parabacteroides	9.43	-2.90	0.00	0.03	Sb.P - Sc
g_Romboutsia	11.19	2.76	0.00	0.04	ENT - ENT3
g_Romboutsia	11.19	2.68	0.00	0.03	ENT - PBS
g_RUG13077	11.64	2.94	0.00	0.02	ENT - PBS
g_RUG13077	11.64	2.61	0.00	0.03	ENT - Sc
g_Schaedlerella	15.30	2.42	0.01	0.03	ENT - ENT3
g_Schaedlerella	15.30	2.16	0.02	0.04	ENT - PBS
g_Schaedlerella	15.30	-3.01	0.00	0.02	ENT3 - Sb.P
g_Schaedlerella	15.30	-2.73	0.00	0.02	PBS - Sb.P
g_Schaedlerella	15.30	2.45	0.01	0.04	Sb.P - Sc
g_Schaedlerella	15.30	2.19	0.01	0.04	Sb.P - SDH1
g_Senegalimassilia	19.50	2.88	0.00	0.01	ENT - PBS
g_Senegalimassilia	19.50	-2.37	0.01	0.03	ENT3 - Sb.P
g_Senegalimassilia	19.50	-3.93	0.00	0.00	PBS - Sb.P
g_Senegalimassilia	19.50	2.74	0.00	0.01	Sb.P - Sc
g_Senegalimassilia	19.50	2.77	0.00	0.01	Sb.P - SDH1
g_Soleaferrea	9.77	-2.71	0.00	0.05	PBS - Sc
g_UBA3402	11.91	-2.55	0.01	0.04	ENT - PBS
g_UBA3402	11.91	-3.05	0.00	0.02	ENT3 - PBS
uc_c_Clostridia	13.57	2.38	0.01	0.04	ENT - ENT3
uc_c_Clostridia	13.57	-3.20	0.00	0.01	ENT3 - Sb.P
uc_c_Clostridia	13.57	-2.55	0.01	0.04	PBS - Sb.P
uc_f_CAG-508	16.90	2.32	0.01	0.04	ENT - ENT3
uc_f_CAG-508	16.90	3.38	0.00	0.01	ENT - PBS
uc_f_CAG-508	16.90	3.05	0.00	0.01	ENT - SDH1
uc_f_CAG-508	16.90	-2.35	0.01	0.05	PBS - Sb.P
uc_f_CAG-508	16.90	-2.18	0.01	0.04	PBS - Sc
uc_f_Muribaculaceae	19.97	3.42	0.00	0.00	ENT - PBS
uc_f_Muribaculaceae	19.97	3.17	0.00	0.00	ENT3 - PBS
uc_f_Muribaculaceae	19.97	-2.53	0.01	0.02	PBS - Sc
uc_f_Muribaculaceae	19.97	-3.94	0.00	0.00	PBS - SDH1
uc_f_Muribaculaceae	19.97	-2.19	0.01	0.04	Sb.P - SDH1

**Taxa end**	**chi2**	**z**	**p**	**Adj.p**	**Comparisons**
g_Akkermansia	12.98	2.82	0.00	0.04	ENT3 - PBS
g_Akkermansia	12.98	2.25	0.01	0.05	ENT3 - Sb.P
g_Akkermansia	12.98	-2.57	0.01	0.04	PBS - Sc
g_Bacteroides	16.49	-3.67	0.00	0.00	PBS - Sb.P
g_Bacteroides	16.49	-2.67	0.00	0.02	PBS - SDH1
g_Bacteroides	16.49	2.72	0.00	0.02	Sb.P - Sc
g_CAG-1031	11.80	2.61	0.00	0.03	ENT - PBS
g_CAG-1031	11.80	2.45	0.01	0.04	ENT3 - PBS
g_CAG-1031	11.80	-2.98	0.00	0.02	PBS - Sb.P
g_Duncaniella	14.14	2.28	0.01	0.03	ENT - PBS
g_Duncaniella	14.14	2.34	0.01	0.03	ENT - Sc

g_Duncaniella	14.14	2.48	0.01	0.03	ENT3 - PBS
g_Duncaniella	14.14	-2.41	0.01	0.03	PBS - Sb.P
g_Duncaniella	14.14	2.48	0.01	0.05	Sb.P - Sc
g_Enterocloster	10.61	-2.75	0.00	0.02	ENT - PBS
g_Enterocloster	10.61	-2.76	0.00	0.04	ENT3 - PBS
g_Enterocloster	10.61	2.25	0.01	0.05	PBS - Sc
g_Enterocloster	10.61	2.41	0.01	0.04	PBS - SDH1
g_Lactobacillus	13.81	3.06	0.00	0.02	ENT3 - Sc
g_Lactobacillus	13.81	2.62	0.00	0.03	ENT3 - SDH1
g_Limosilactobacillus	19.03	2.67	0.00	0.02	ENT - Sc
g_Limosilactobacillus	19.03	2.03	0.02	0.05	ENT - SDH1
g_Limosilactobacillus	19.03	2.28	0.01	0.03	ENT3 - PBS
g_Limosilactobacillus	19.03	3.31	0.00	0.01	ENT3 - Sc
g_Limosilactobacillus	19.03	2.66	0.00	0.01	ENT3 - SDH1
g_Limosilactobacillus	19.03	2.94	0.00	0.01	Sb.P - Sc
g_Limosilactobacillus	19.03	2.27	0.01	0.03	Sb.P - SDH1
g_Muribaculum	14.65	-2.82	0.00	0.02	PBS - Sb.P
g_Muribaculum	14.65	3.11	0.00	0.01	Sb.P - Sc
g_Oscillibacter	10.15	-2.89	0.00	0.03	ENT - Sc
g_Pseudocitrobacter	10.88	2.61	0.00	0.03	ENT3 - PBS
g_Turicibacter	12.51	3.32	0.00	0.01	ENT - ENT3
g_Turicibacter	12.51	-2.66	0.00	0.03	ENT3 - Sc
g_UMGS1872	13.97	-3.54	0.00	0.00	ENT - PBS
g_UMGS1872	13.97	-2.67	0.00	0.03	ENT3 - PBS
g_UMGS1872	13.97	2.53	0.01	0.03	PBS - Sb.P
g_UMGS1872	13.97	2.39	0.01	0.03	PBS - SDH1
uc_f_Muribaculaceae	14.12	2.65	0.00	0.02	ENT - PBS
uc_f_Muribaculaceae	14.12	3.20	0.00	0.01	ENT3 - PBS
uc_f_Muribaculaceae	14.12	-2.23	0.01	0.04	PBS - Sb.P
uc_f_Muribaculaceae	14.12	-2.91	0.00	0.01	PBS - Sc
uc_f_Muribaculaceae	14.12	-3.08	0.00	0.01	PBS - SDH1
uc_o_Bacteroidales	15.91	-3.65	0.00	0.00	PBS - Sb.P
uc_o_Bacteroidales	15.91	-2.79	0.00	0.02	PBS - Sc

Supplementary Table S4. Results of Wilcoxon test comparing the alpha-diversity defined by observed-richness of the inflamed condition with their respective healthy controls receiving the same treatment at the different timepoints.

Comparison	Pre (*p*)	Mid (*p*)	End (*p*)
PBS vs DSS+PBS	1.00	1.00	0.049
Sc vs DSS+Sc	0.47	0.0003	0.71
SDH1 vs DSS+SDH1	0.77	0.037	0.04
ENT vs DSS+ENT	0.85	0.015	0.037
Sb.P vs DSS+Sb.P	0.08	0.005	0.0048
ENT3 vs DSS+ENT3	0.41	0.93	0.8

1.2 Supplementary figures

Supplementary Figure S1. PCA-plots transcriptomic analysis of colon tissue (A) effect of inflammation, (B) cage-effect and (C) effect of condition (inflammation and treatment).

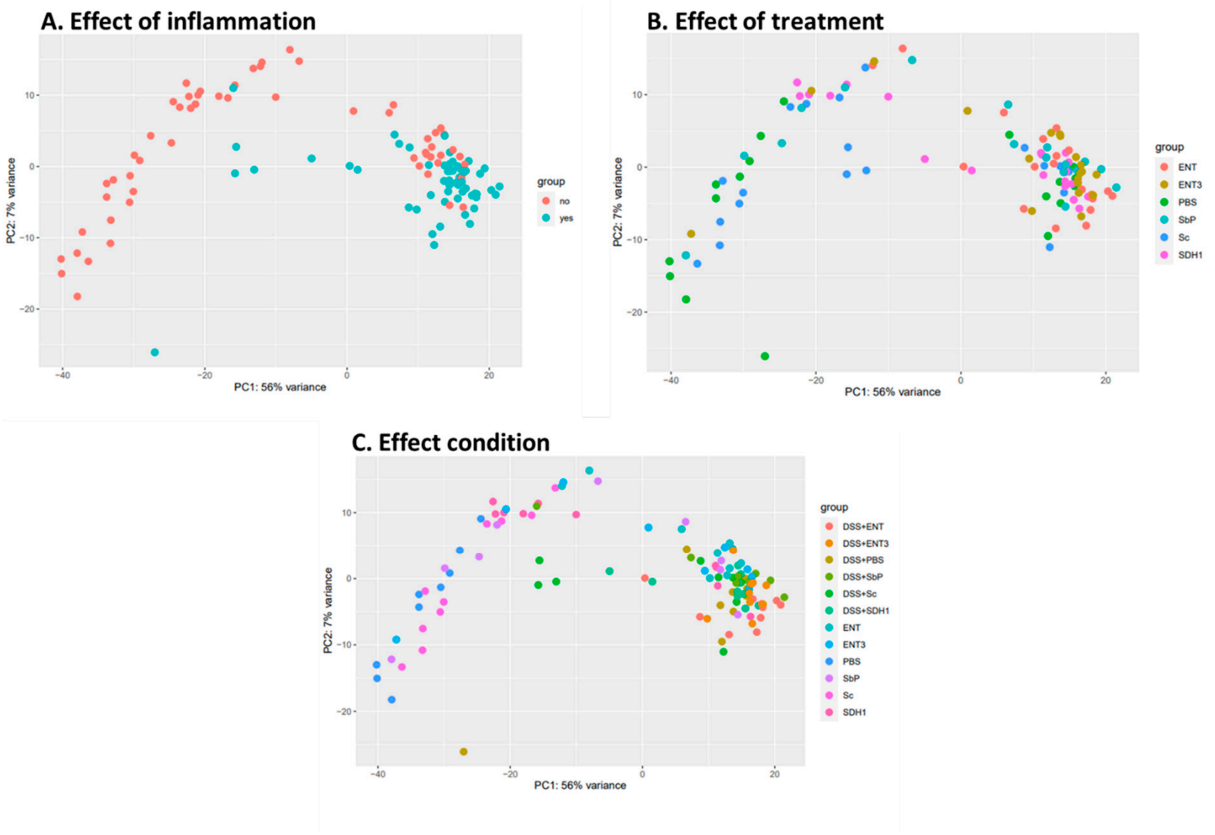

Supplementary Figure S2. Observed richness (alpha diversity) in inflamed subgroups at the different timepoints. (A) Baseline (B) Mid experiment (C) End experiment.

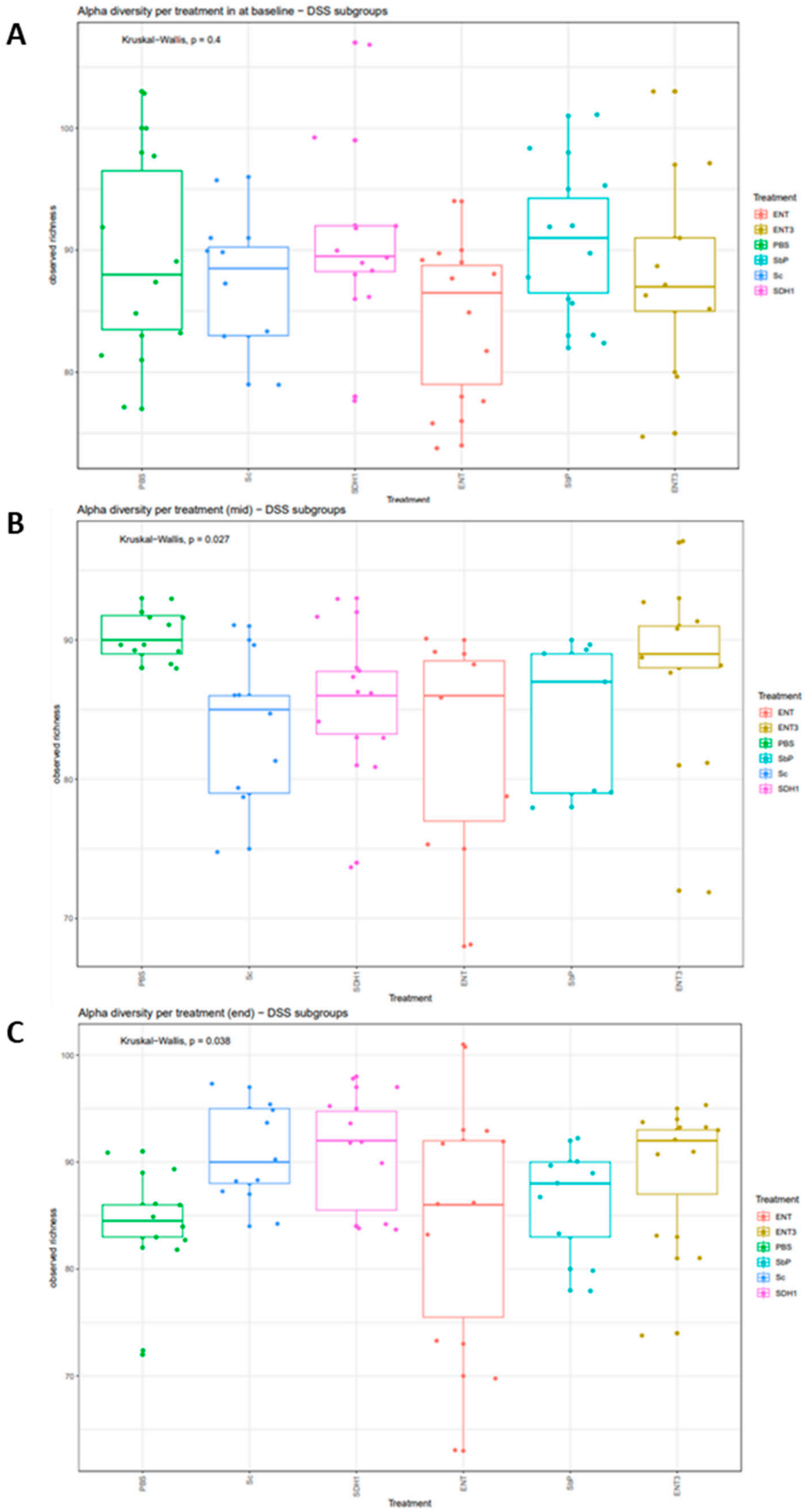

Supplementary Figure S3. Observed richness (alpha diversity) within treatment and timepoint.

A. PBS

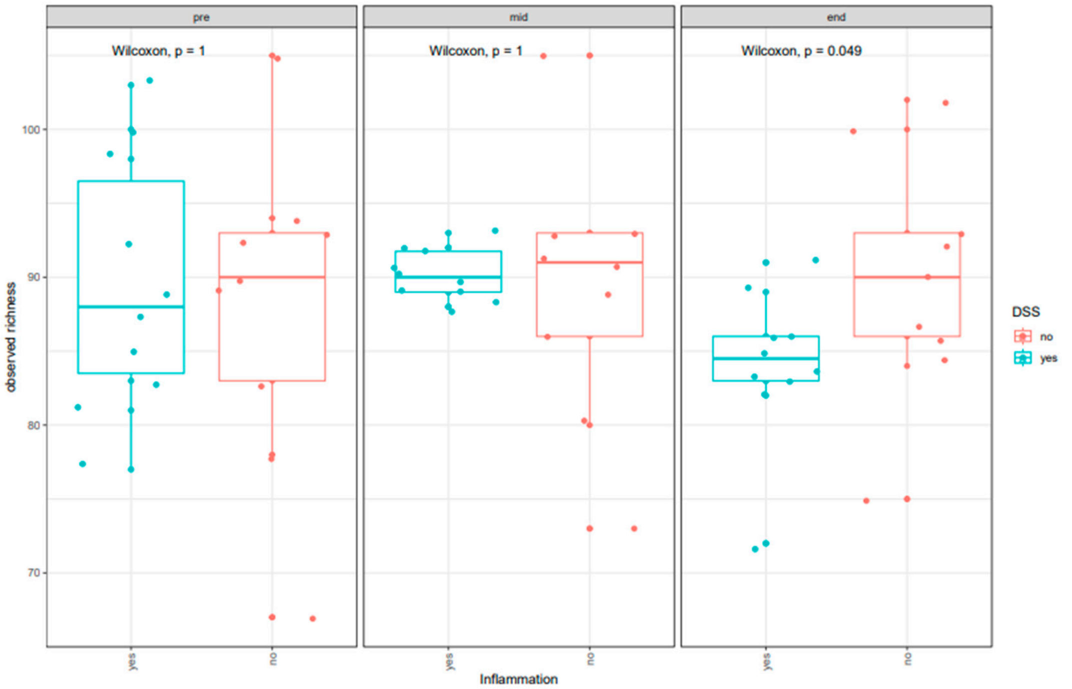

B. Sc

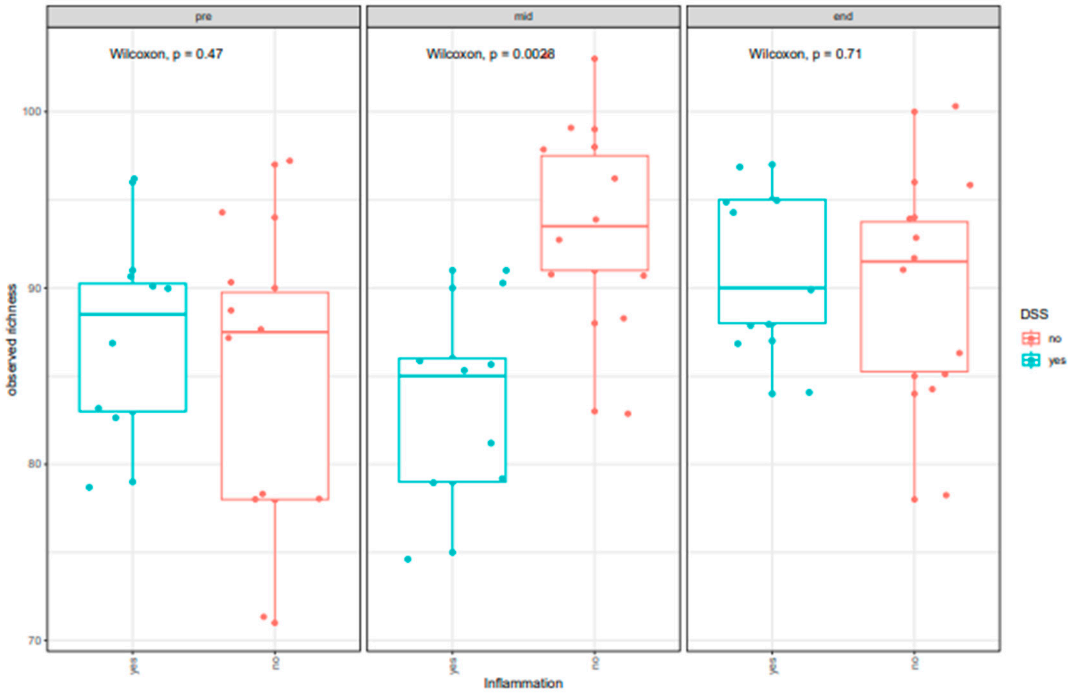

C. SDH1

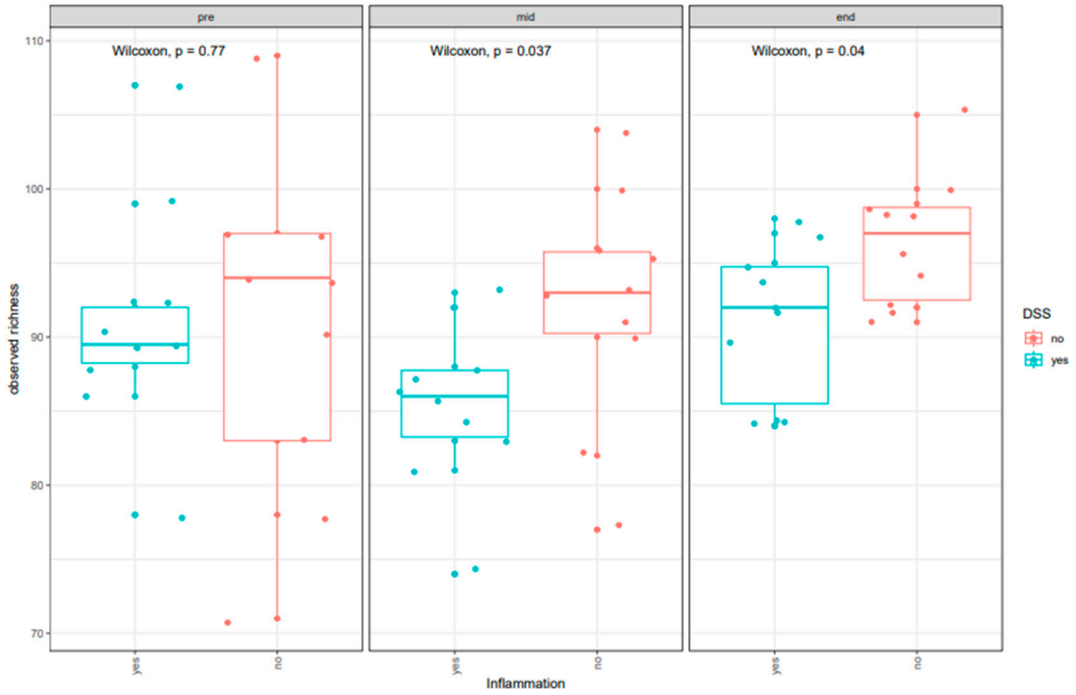
pre
mid
end
Wilcoxon, p = 0.77
Wilcoxon, p = 0.037
Wilcoxon, p = 0.04
110
100
90
80
70
observed richness
yes
no
Inflammation
DSS
no
yes

D. ENT

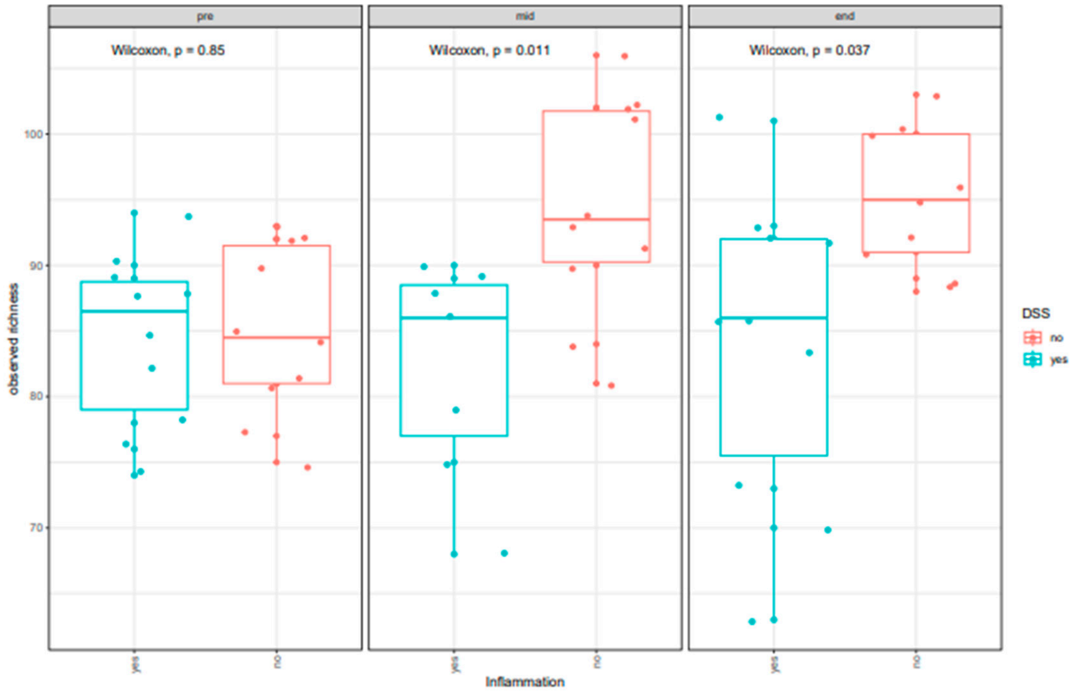
pre
mid
end
Wilcoxon, p = 0.85
Wilcoxon, p = 0.011
Wilcoxon, p = 0.037
100
90
80
70
observed richness
yes
no
Inflammation
DSS
no
yes

E. Sb.P

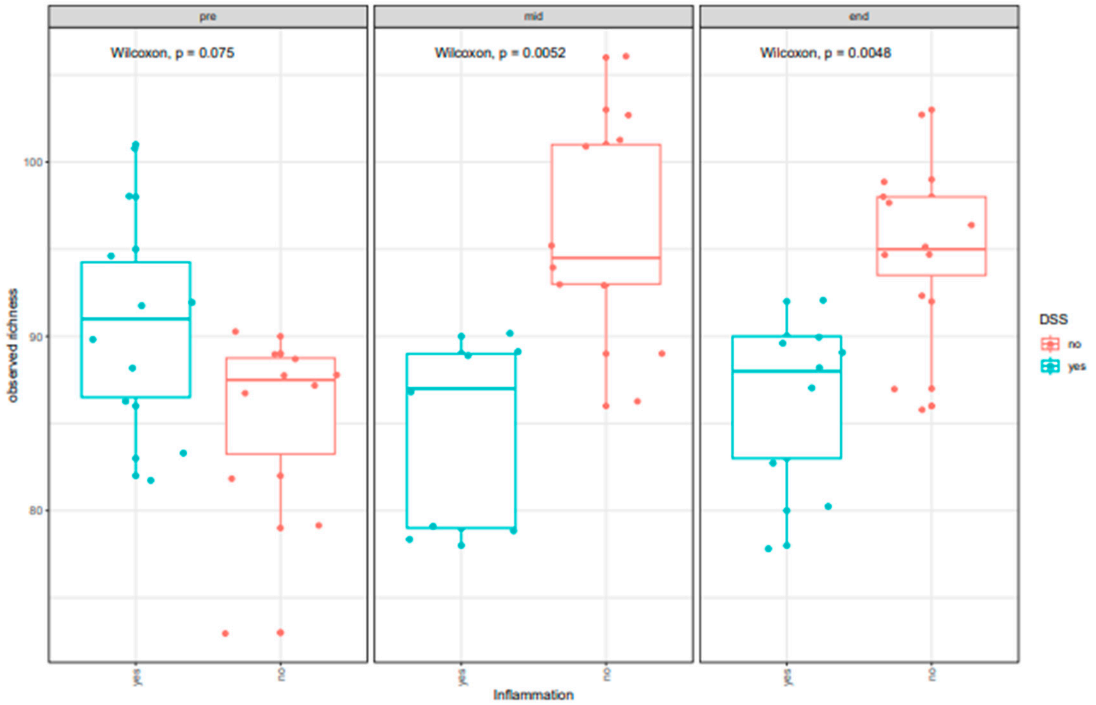
pre
mid
end
Wilcoxon, p = 0.075
Wilcoxon, p = 0.0052
Wilcoxon, p = 0.0048
observed richness
100
90
80
yes
no
Inflammation
DSS
no
yes

F. ENT3

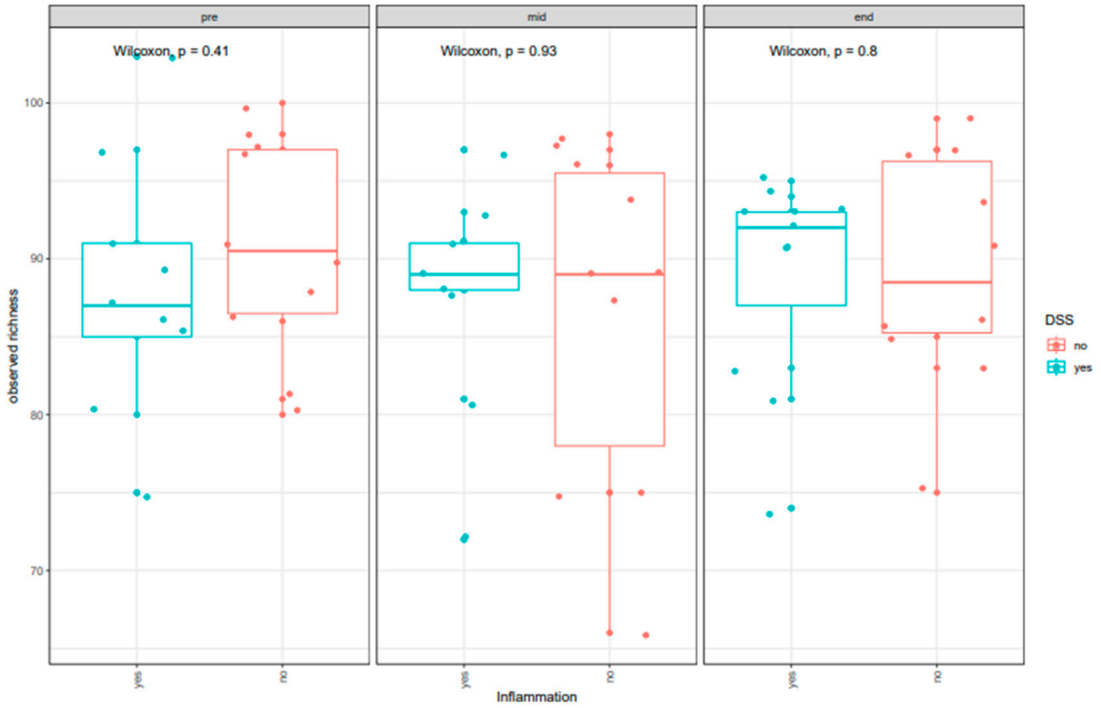
pre
mid
end
Wilcoxon, p = 0.41
Wilcoxon, p = 0.93
Wilcoxon, p = 0.8
observed richness
100
90
80
70
yes
no
Inflammation
DSS
no
yes

Supplementary Figure S4. Observed richness (alpha diversity) in healthy subgroups at the different timepoints. (A) Baseline (B) Mid experiment (C) End experiment.

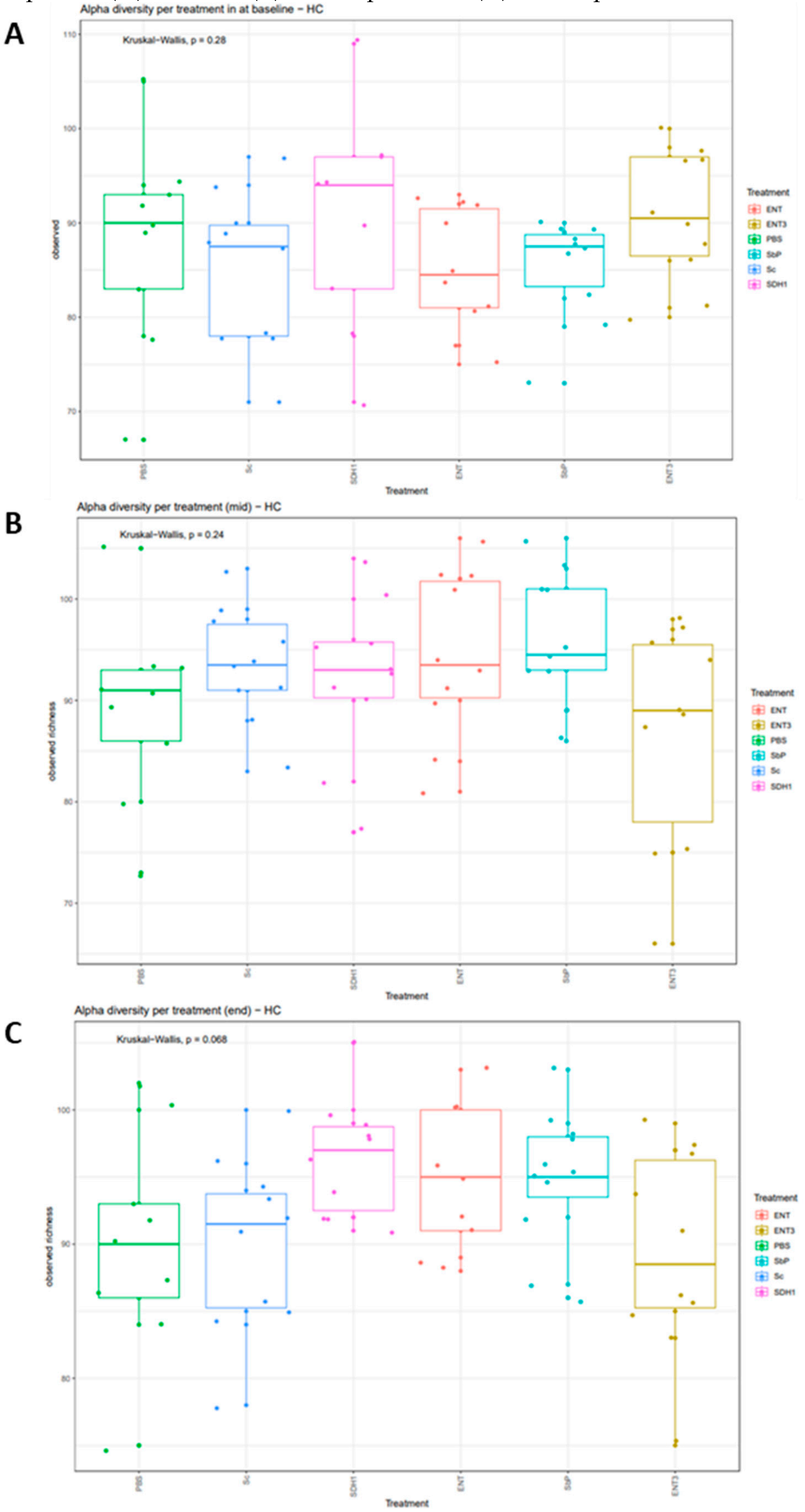

Supplement: Supplementary file 1 [file nutrients-16-02668-s001.zip › nutrients-3111409-supplementary.pdf]
